# HOXD10 regulates intestinal permeability and inhibits inflammation of dextran sulfate sodium-induced ulcerative colitis through the inactivation of the Rho/ROCK/MMPs axis

**DOI:** 10.1515/med-2023-0844

**Published:** 2024-05-13

**Authors:** Jing Xu, Nana Lin

**Affiliations:** Department of Geriatrics, Affiliated Hangzhou First People’s Hospital, School of Medicine, Westlake University, No. 469, Shenban Road, Gongshu District, Hangzhou, Zhejiang, 310000, China; Department of Geriatrics, Affiliated Hangzhou First People’s Hospital, School of Medicine, Westlake University, Hangzhou, Zhejiang, 310000, China

**Keywords:** ulcerative colitis, HOXD10, inflammatory response, intestinal barrier function, Rho/ROCK/MMPs axis

## Abstract

Ulcerative colitis (UC) has been identified as a severe inflammatory disease with significantly increased incidence across the world. The detailed role and mechanism of HOXD10 in UC remain unclear. In present study, we found that HOXD10 was lowly expressed in UC samples and was notably decreased by dextran sulfate sodium (DSS) administration. Overexpression of HOXD10 dramatically ameliorated DSS-induced UC symptoms, including the loss of weight, increased disease activity index values, and the shortened colon length. Additionally, terminal-deoxynucleoitidyl transferase mediated nick end labeling and immunohistochemistry staining assays showed that HOXD10 overexpression suppressed cell apoptosis and facilitated proliferation of colon tissues after DSS treatment. Moreover, HOXD10 overexpression obviously suppressed DSS-triggered inflammatory response by decreasing the expression level of TNF-α, IL-6, and IL-1β. Furthermore, overexpression of HOXD10 effectively restored the intestinal permeability, thereby alleviating DSS-induced intestinal barrier dysfunction. Mechanistic study demonstrated that HOXD10 significantly reduced the activities of Rho/ROCK/MMPs axis in colon tissues of mice with UC. In conclusion, this study revealed that HOXD10 might effectively improve DSS-induced UC symptoms by suppressing the activation of Rho/ROCK/MMPs pathway.

## 1 Introduction

Ulcerative colitis (UC) is a chronic, nonspecific inflammatory disease, that can harm rectum and colon and frequently relapses [1,2]. Abdominal pain and bloody diarrhea are the typical symptoms of UC, which can be diagnosed via colonoscopy and colon biopsy [3,4]. In recent years, the incidence of UC has increased worldwide. In addition, the leakage of the intestinal epithelial barrier and the decreased intestinal permeability are important pathological features of UC [5,6]. Extracellular mucus and tight junctions are considered as vital components of mucosal barrier, protecting against harmful substances in the intestinal cavity. It has been reported that the release of UC submucosal inflammatory factors destroys the structural integrity of intestinal mucosal barrier, contributing to changes in intestinal mucosal permeability, thereby exacerbating abnormal submucosal immune responses [7,8]. Therefore, it is essential to explore the potential targeted genes that modulate intestinal permeability and inflammation in UC.

HOXD10, a member of the human homeobox (HOX) gene family, is closely associated with cell differentiation and morphogenetic embryo development [9]. Previous study illustrated that HOXD10 depletion down-regulates the activity and migration of RAFLS in rheumatoid arthritis and ameliorates arthritis by inhibiting p38/c-Jun signaling pathway [9]. Ruan et al. discovered that HOXD10 exerts anti-inflammatory and neuroprotective effects. HOXD10 overexpression suppresses neuronal apoptosis, inflammatory response, and oxidative stress, thereby restoring cognitive deficits in Alzheimer’s disease mice [10]. Besides, HOXD10 also plays crucial roles in tumorigenesis. For example, HOXD10 is identified as a tumor suppressor gene to inhibit cancer development and enhance apoptosis in human cholangiocarcinoma [11]. Down-regulation of HOXD10 facilitates endometrial carcinoma progression and aggravates its malignant phenotype [12]. However, the role and mechanism of HOXD10 in UC are still unclear. Data analysis of GEO chip (GSE48634) indicated that HOXD10 was significantly low expressed in the colon tissues of UC patients. Therefore, we speculated that HOXD10 plays an important role in UC.

Emerging evidence reported that Rho/ROCK signaling pathway plays a vital role in various inflammatory response, such as in rheumatoid arthritis [13] and non-specific neuroinflammation [14]. In addition, it is reported that the Rho/ROCK inactivation protects the integrity of the epithelial barrier induced by dextran sulfate sodium (DSS), relieves oxidative stress, and inhibits the expression of inflammatory mediators and pro-inflammatory cytokines [15]. Zhang et al. discovered that sishen improves UC by modulating the Rho/ROCK axis [16]. Moreover, matrix metalloproteinases (MMPs) are also associated with the decreased intestinal tissue tight junction protein Claudin-5. The increased expression of MMP-2 and MMP-9 has been shown to enhance intestinal mucosal permeability, suggesting that the expression of MMPs promotes the destruction of epithelial barrier and aggravates the symptoms of UC [17,18]. Interestingly, it is reported that Rho/ROCK signaling regulates the expression of MMPs in diverse human diseases [19]. For example, Rho/ROCK inactivation reduces the expression of MMPs in hepatocellular carcinoma [20]. Based on these findings, the inactivation of Rho/ROCK/MMPs cascade signals may be critical to mitigate UC development. However, few studies have revealed the upstream genes that regulate Rho/ROCK/MMPs axis during UC progression. Notably, previous study proved that HOXD10 is capable of inhibiting the activity of Rho/ROCK axis [10,11]. Nevertheless, whether HOXD10 regulates the Rho/ROCK/MMP pathway in UC remains unclear.

This study for the first time revealed that HOXD10 reduced inflammatory response and apoptosis of intestinal tissue cells, and ameliorated intestinal barrier function by modulating Rho/ROCK/MMPs axis, thereby increasing intestinal permeability in DDS-induced UC mice model.

## 2 Materials and methods

### 2.1 Animals treatment

The 24 C57BL/6 male mice were obtained from Chengdu Dasuo Experimental Animal Co., Ltd (China) and were kept in animal facilities at controlled environment (23 ± 3°C, 60 ± 15% humidity). Additionally, all mice had free access to the same food and water. All experiments were supported by the Animal Ethical Committee of Affiliated Hangzhou First People’s Hospital.

### 2.2 Induction of UC

The 24 C57BL/6 male mice aged 8–10 weeks were divided into four groups with six mice in each group: sham + AAV-NC group, sham + AAV-HOXD10 group, DSS + AAV-NC group, and DSS + AAV-HOXD10 group. The mice in DSS + AAV-NC and DSS + AAV-HOXD10 group were treated with 4% DSS (9011-18-1, Guangxi Qili Pharmaceutical Co. Ltd, China), while the mice in sham + AAV-NC and sham + AAV-HOXD10 group were treated with water without DSS. To investigate the effects of HOXD10 on UC, 1 × 10^11^ vg adeno-associated virus 9 (AAV-9) particles carrying HOXD10 or its scramble control obtained from Han Hang Seng Technology (Shanghai) Co., Ltd (China) were injected into each mouse in the indicated group (sham + AAV-HOXD10 and DSS + AAV-HOXD10 group or sham + AAV-NC and DSS + AAV-NC group, respectively) through tail vein and the UC model was established 2 weeks later. The body weight, diarrhea, and bleeding were observed and recorded daily after the modeling began. Then the disease activity index (DAI) was estimated based on the data of body weight loss, stool occult blood positivity, or hemorrhoea as well as stool consistency as described in previous reports [8,21]. After 7 days, mice blood from caudal vein was harvested and centrifuged at 12,000 rpm for 5 min for subsequent analyses. Then the mice were sacrificed for isolating colon tissues. The length from the cecum to the anus was measured and the colon tissues were preserved in liquid nitrogen for next experiments.

### 2.3 Bioinformatics analysis

The data were collected from the GEO database (https://www.ncbi.nlm.nih.gov/geo/). The expression profiling data of GSE48634 chip was analyzed using GEO2R platform to reveal the potential genes in UC. The screening criteria were *p* < 0.05, log2 FC (fold change) >1.5, or log2 FC < − 1.5 [22]. The filtering information of GSE48634 chip was as described in previous report [23]. Briefly, the GSE48634 chip contains 68 patients diagnosed with UC and 69 healthy controls (collected from the UC microarrays without disease).

### 2.4 Quantitative real time polymerase chain reaction (qPCR)

Total RNA of colon tissues was extracted employing Beyozol reagent (R0011, Beyotime, China) following the manufacturer’s instruction. A BeyoRT™ First Strand cDNA Synthesis Kit (D7166, Beyotime, China) was utilized to produce the complementary DNA according to the protocol of the kit. Then the qRT-PCR was conducted with MonAmp™ Taqman qPCR Mix (MQ30101S, MQ30101S, Monad, China) on a fluorescence quantitative PCR instrument (CFX96, Bio-rad, USA). The relative expression level of targeted genes was analyzed using 2^–ΔΔCt^ method and β-actin was regarded as the control gene. All sequences of primers are provided in Table 1.

**Table 1:** Sequences of qRT-PCR primers

Gene	Forward	Reverse
HOXD10	5′-GACATGGGGACCTATGGAATGC-3′	5′-CGGATCTGTCCAACTGTCTACT-3′
IL-6	5′-CTGCAAGAGACTTCCATCCAG-3′	5′-AGTGGTATAGACAGGTCTGTTGG-3′
IL-1β	5′-GCAACTGTTCCTGAACTCAACT-3′	5′-ATCTTTTGGGGTCCGTCAACT-3′
TNF-α	5′-AGAACTCCAGGCGGTGTCT-3′	5′-TCCCTCAGGGGTGTCCTTAG-3′
β-actin	5′-AACCCTAAGGCCAACCGTGAAAAG-3′	5′-GCTCGAAGTCTAGGGCAACATA-3′

### 2.5 Western blotting

Colon tissues were lysed using pre-chilled RIPA lysis buffer (P0013D, Beyotime, China) and then were centrifuged (12,000 rpm, 15 min) at 4°C for harvesting total protein. Then the concentration of protein was determined utilizing BCA Protein Quantification Kit BCA (20201ES76; YESEN, China) according to the protocol of kit. Next, total protein from tissues were separated with SDS-PAGE and transferred onto polyvinylidene fluoride membranes (PW60101S; Monad, China). The membranes were blocked using 5% bovine serum albumin (Standard Grade, 36101ES25; YESEN, China) dissolving in tris borate saline with 0.1% tween 20. After blocking, the membranes were incubated with specific primary antibodies, anti-HOXD10 antibody (1:1,000, ab138508; Abcam, UK), anti-BAX-antibody (1:1,000, ab32503; Abcam, UK), Bcl-2 rabbit mAb (1:1,000, A19693; Abclonal, China), anti-cleaved caspase-3 antibody (1:1,000, ab2302; Abclonal, China), caspase-3 rabbit pAb (1:1,000, A2156; Abclonal, China), phospho-NF-κB p65 (Ser536) (93H1) rabbit mAb (1:1,000, 3033; Cell Signaling Technology, USA), MUC2 rabbit mAb (1:1,000, A4767; Abclonal, China), anti-Claudin 3 antibody (1:1,000, ab15102; Abcam, UK), Occludin polyclonal antibody (1:1,000, 71-1500; Invitrogen Antibodies, USA), ZO-1 rabbit pAb (1:1,000, A0659; Abclonal, China), RhoA rabbit mAb (1:1,000, A19106; Abclonal, China), Rac1/Cdc42 antibody (1:1,000, 4651; Cell Signaling Technology, USA), ROCK1 rabbit mAb (1:1,000, A11158; Abclonal, China), ROCK2 rabbit mAb (1:1,000, A2395; Abclonal, China), MMP2 rabbit mAb (1:1,000, A19080; Abclonal, China), MMP2 rabbit mAb (1:1,000, A19080; Abclonal, China), or β-actin rabbit mAb (1:1,000, AC038; Abclonal, China) overnight at 4°C. Finally, the membranes were incubated with goat anti-rabbit IgG H&L (HRP) (1:5,000, ab6721; Abcam, UK) and the protein bands were detected utilizing Super ECL Detection Reagent ECL kit (36208ES60; YESEN, China) on a Hesper chemiluminescence imaging system (GD50401; Monad, China). The relative expression of targeted protein was analyzed with Image Lab software [24].

### 2.6 Immunofluorescence staining

The immunofluorescence staining was performed as described in previous study [25]. Briefly, the colon tissue sections (4 μm thick) embedded in paraffin are dewaxed and rehydrated. Then the sections were incubated with primary antibody anti-HOXD10 antibody (1:50, ab138508; Abcam, UK), MUC2 rabbit mAb (1:1,000, A4767; Abclonal, China), Occludin polyclonal antibody (1:1,000, 71-1500; Invitrogen Antibodies, USA), or ZO-1 rabbit pAb (1:1,000, A0659; Abclonal, China) overnight. After washing three times using phosphate buffer saline (PBS; C0221A, Beyotime, China), the sections were treated with biotin-labeled goat anti-rabbit IgG (H+L) with high mole ratio (1:200, A0279; Beyotime, China) for 1 h, followed by incubating with fluorescein diacetate (1:50, 40720ES03; YESEN, China) for another 1 h. Next, the sections were stained with 2-(4-amidinophenyl)-6-indolecarbamidine dihydrochloride (C1002; Beyotime, China) for 30 min. Finally, the images of random six fields were obtained employing a fluorescence microscope (BX53FL, Olympus, Japan).

### 2.7 Hematoxylin & eosin (H&E) staining

The colon tissues were fixed using 4% paraformaldehyde fix solution (P0099; Beyotime, China) and embedded in paraffin, followed by cutting into 4 μm sections with a freezing microtome (FS800; Shenzhen Rayward Life Technology Co., Ltd, China). Next, a hematoxylin and eosin staining kit (C0105M, Beyotime, China) was employed to stain the sections following the kit’s protocol. Finally, the histological analysis of the tissue slices was performed utilizing an optical microscope (THUNDER Imager Tissue, Leica, Germany).

### 2.8 Terminal-deoxynucleoitidyl transferase mediated nick end labeling (TUNEL) assay

Cell apoptosis in colon tissues were examined by employing a TUNEL Apoptosis Detection Kit (40308ES20; YESEN, China) as described in previous report [26]. Briefly, colon tissue sections (4 μm thick) were treated with PBS containing 1% endopeptidase K (ST535; Beyotime, China) for 15 min. Then the enzyme and label reagent were added to the tissue sections in a ratio of 1:9, followed by treating the sections with 50 μL of converter-POD for 30 min. Finally, the images of random six fields were observed with an optical microscope (THUNDER Imager Tissue, Leica, Germany).

### 2.9 Immunohistochemistry (IHC) staining

IHC staining was introduced to determine the ki-67 expression in colon tissues. The tissues (4 μm thick) were fixed using 4% paraformaldehyde fix solution (P0099; Beyotime, China) and embedded in paraffin, followed by cutting into 4 μm sections with a freezing microtome (FS800, Shenzhen Rayward Life Technology Co., Ltd, China). Ki-67 rabbit mAb (A11005) and biotin-labeled goat anti-rabbit IgG (H+L) were incubated with sections successively according to the procedure described in previous report [27]. Finally, the images of sections were observed with an optical microscope (THUNDER Imager Tissue, Leica, Germany).

### 2.10 Enzyme-linked immunosorbent assay (ELISA)

The expression level of TNF-α, IL-6, and IL-1β in serum was detected using mouse TNF-α (tumor necrosis factor alpha) ELISA Kit (E-EL-M3063, Elabscience, China), mouse IL-6 (interleukin 6) ELISA Kit (E-EL-M0044c, Elabscience, China), or mouse IL-1β (interleukin 1 beta) ELISA Kit (E-EL-M0037c, Elabscience, China) according to the manufacturer’s instructions, respectively.

### 2.11 Intestinal barrier function analysis

For measurement of FITC-dextran, mice were given FITC-dextran (MW: 4 kD, HY-128868A, MCE, USA) by gavage administration after fasting for 4 h. After another 4 h, blood was collected from caudal vein and plasma by centrifugation at 4°C (12,000 rpm, 10 min). Then the plasma was diluted with PBS, and the content of FITC-dextran in serum was detected by a fluorescence spectrophotometer (933N, Ditu (Shanghai) Biotechnology Co., Ltd, China) at excitation wavelength of 485 nm and emission wavelength of 525 nm. The concentration of FITC-dextran in untreated serum diluted with PBS was used to draw the standard curve.

### 2.12 Period-Schiff (PAS) staining

The goblet cells loss was determined by performing PAS staining analysis on colon tissues. The experiments were performed using a Periodic Acid-Schiff Staining Kit (C0142S, Beyotime, China) according to the manufacturer’s protocol. Finally, the images of sections (4 μm thick) were observed with an optical microscope (THUNDER Imager Tissue, Leica, Germany).

### 2.13 Statistical analysis

All the data were presented as the mean ± SD and the statistical analyses were conducted by Graphpad Prism 8.0. Student’s *t*-test was employed for two group comparisons, while one-way ANOVA with Bonferroni post-test was introduced for multiple group comparisons. The *p* ＜ 0.05 was regarded as statistically significant. All the experiments were repeated three times.

## 3 Results

### 3.1 HOXD10 is down-regulated in UC

**Figure 1:**
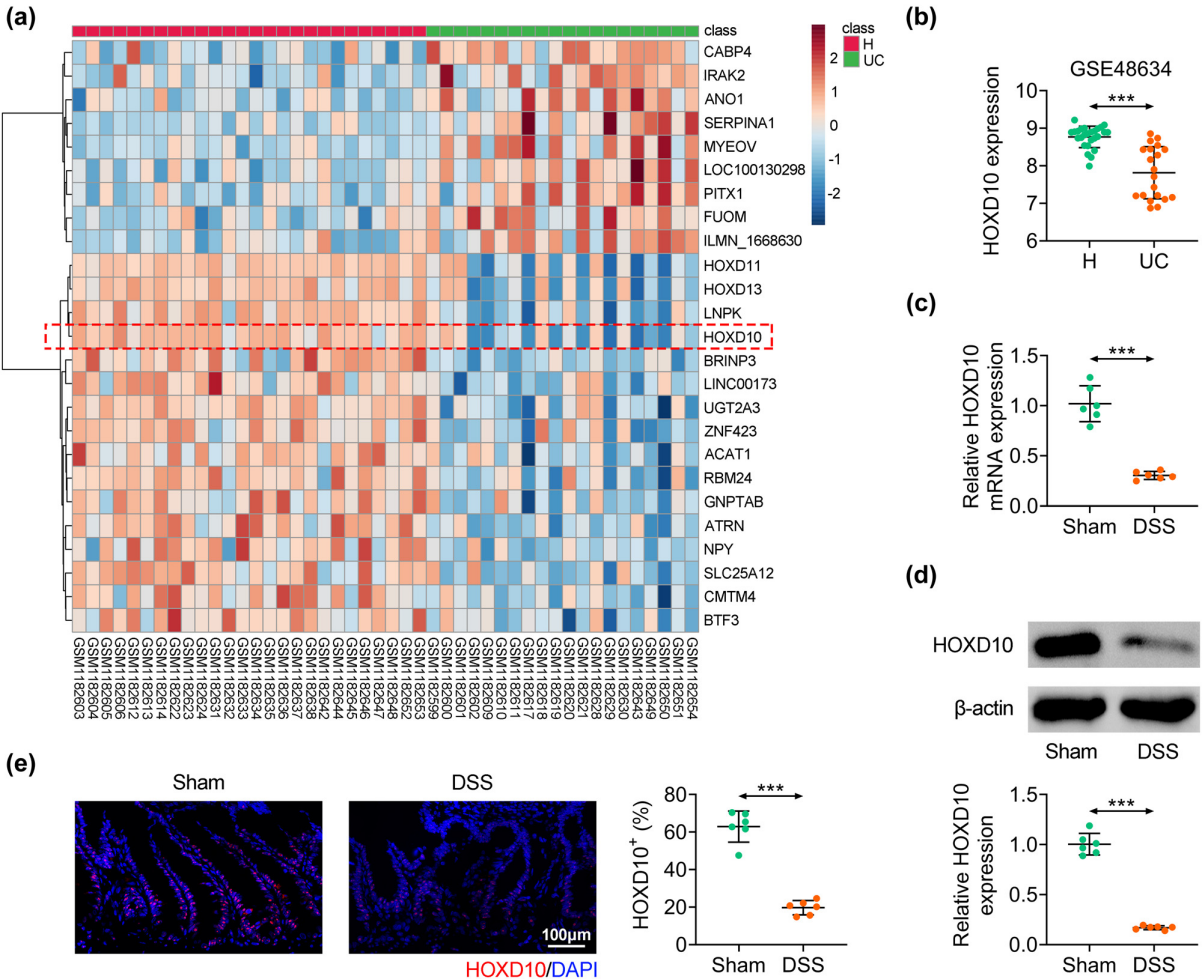
HOXD10 is down-regulated in UC. (a) The genes expression profiles exhibited as the manner of heatmap. *X*-axis: samples; *Y*-axis: genes. Red: high expression; blue: low expression. (b) The expression level of HOXD10 obtained by analyzing the data from GSE48634 chip (^***^ *p* < 0.001). (c) The mRNA level of HOXD10 detected via qRT-PCR analysis (^***^ *p* < 0.001, C57BL/6 male mice, *n* = 6/group). (d) The protein level of HOXD10 detected via western blotting analysis (^***^ *p* < 0.001, C57BL/6 male mice, *n* = 6/group). (e) The expression level of HOXD10 detected by immunofluorescence analysis (^***^ *p* < 0.001, C57BL/6 male mice, *n* = 6/group). Student’s *t*-test was employed for the statistical analysis in two groups.

A microarray was performed to compare the expression profile of genes in healthy control samples and UC samples from GSE48634 chip. The data illustrated that HOXD10 was notably low expressed in UC samples (Figure 1a and b). To confirm the expression level of HOXD10 in UC, the C57BL/6 mice were employed to induce UC by administrating DSS. As shown in Figure 1c and d, the expression of HOXD10 in colon tissues was markedly decreased in DSS group compared with sham group (Figure 1c and d). Moreover, immunofluorescence staining of HOXD10 proved that HOXD10-positive cells in colon tissues of mice were dramatically reduced in DSS group (Figure 1e). Taken together, these findings demonstrated that HOXD10 expression was decreased in UC, suggesting that HOXD10 might be associated with the progression of UC.

### 3.2 HOXD10 overexpression relieves DSS-induced UC

**Figure 2:**
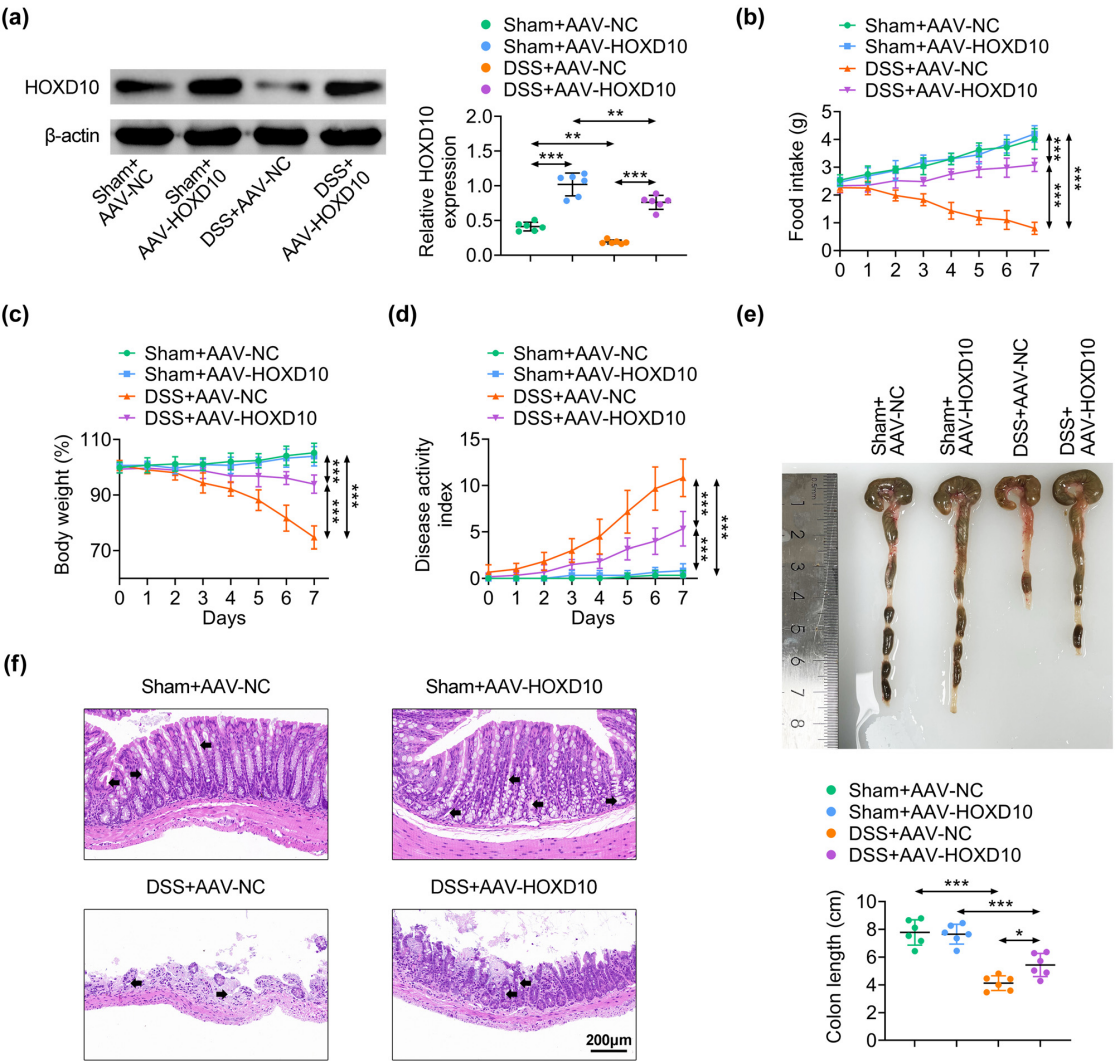
HOXD10 overexpression relieves DSS-induced UC. (a) The protein level of HOXD10 detected via western blotting analysis (^***^ *p* < 0.001, ^**^ *p* < 0.01, C57BL/6 male mice, *n* = 6/group). (b) The food intake of mice over a 7-day period (^***^ *p* < 0.001). (c) The body weight of mice in indicated groups (^***^ *p* < 0.001, C57BL/6 male mice, *n* = 6/group). (d) The DAI score of mice in indicated groups (^***^ *p* < 0.001, C57BL/6 male mice, *n* = 6/group). (e) The colon length of mice in indicated groups (^***^ *p* < 0.001, ^*^ *p* < 0.05, C57BL/6 male mice, *n* = 6/group). (f) The H&E staining assay (C57BL/6 male mice, *n* = 6/group). The one-way ANOVA with Bonferroni post-test was introduced for the statistical analysis in multiple groups.

To investigate the roles of HOXD10 in DSS-induced UC, AAV particles carrying scramble sequences (NC) or HOXD10 were used to ectopically express HOXD10. Western blotting analysis indicated that AAV-HOXD10 increased HOXD10 expression in colon tissues and remarkably recovered the level of HOXD10 in DSS mouse model (Figure 2a). Compared with the sham group, DSS induction notably reduced the food intake of mice over a 7-day period. However, HOXD10 overexpression effectively restored the food intake of mice after DSS treatment (Figure 2b). In addition, the DSS treatment caused significant colitis-associated symptoms in mice, including the loss of weight, increase in DAI values, and shortened colon [28]. Compared with mice in AAV-NC group, AAV-HOXD10 dramatically ameliorated the DSS-induced symptoms, with less weight loss, lower DAI scores, and longer colon length (Figure 2c–e). Furthermore, H&E staining assay demonstrated that DSS treatment resulted in severe diffuse destruction of the colon epithelium and extensive infiltration of inflammatory cells in the epithelium and lamina propria, while HOXD10 overexpression efficiently restored these damages (Figure 2f). Collectively, these results proved that HOXD10 reduced the sensitivity of mice to DSS exposure.

### 3.3 HOXD10 overexpression inhibits DSS-induced apoptosis and promotes proliferation of colon tissues

**Figure 3:**
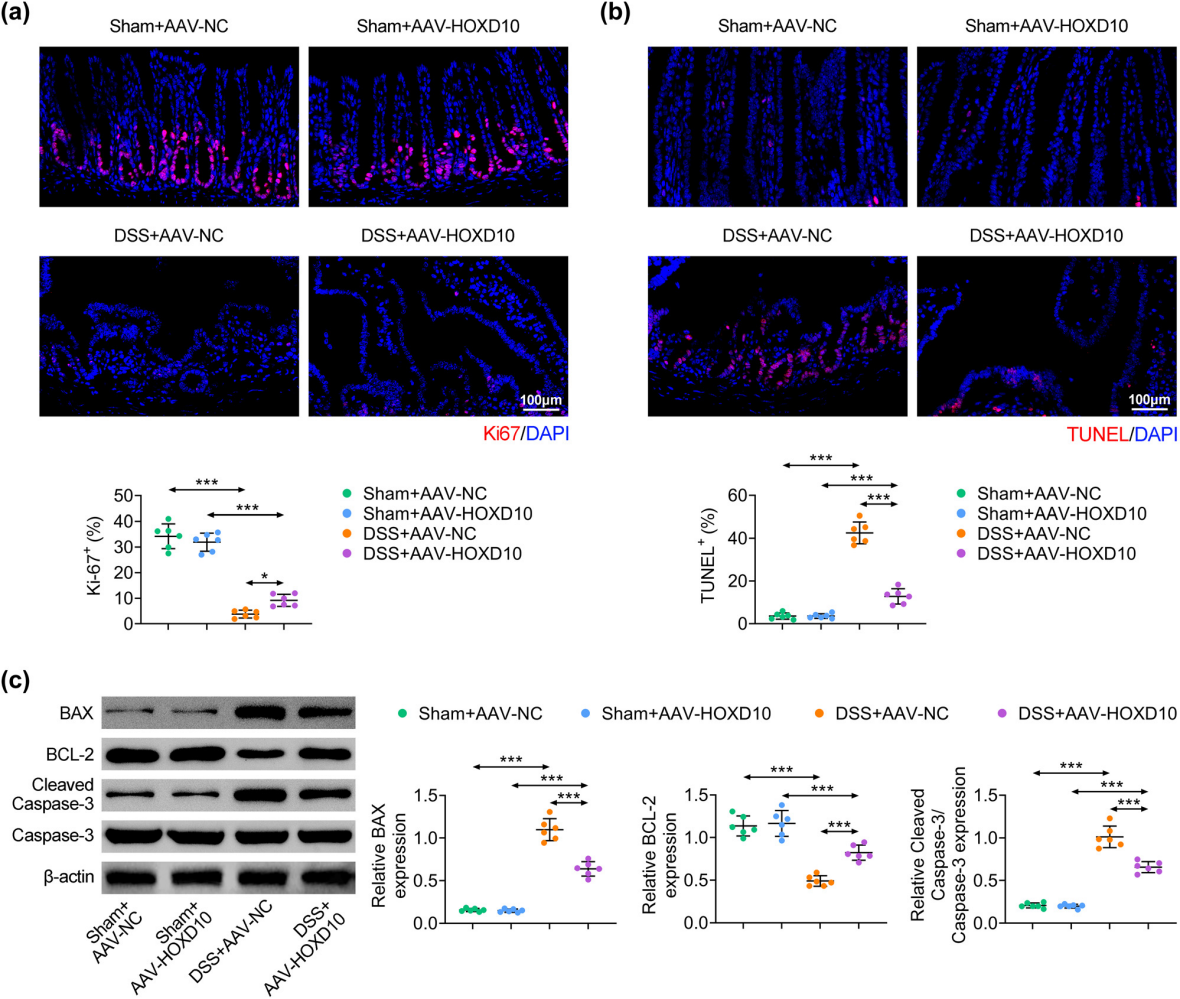
HOXD10 overexpression inhibits DSS-induced apoptosis and promotes proliferation of colon tissues. (a) Cell proliferation in colon tissues detected via ki-67 staining assay (^***^ *p* < 0.001, C57BL/6 male mice, *n* = 6/group). (b) Cell apoptosis in colon tissues detected via TUNEL analysis (^***^ *p* < 0.001, C57BL/6 male mice, *n* = 6/group). (c) The expression of apoptosis-related proteins detected via western blotting analysis (^***^ *p* < 0.001, C57BL/6 male mice, *n* = 6/group). The one-way ANOVA with Bonferroni post-test was introduced for the statistical analysis in multiple groups.

Subsequently, the effects of HOXD10 on cell proliferation and apoptosis of colon tissues were estimated via immunology technique. The ki-67 immunoreactivity and TUNEL assay were introduced to detect cell proliferation and apoptosis in colon tissues, respectively. Ki-67 positive cells were decreased in DSS-treated mice compared with sham mice, which were significantly increased by overexpression of HOXD10 (Figure 3a). Additionally, DSS treatment notably induced cell apoptosis in colon tissues, whereas overexpression of HOXD10 effectively attenuated DSS-induced cell apoptosis (Figure 3b). Moreover, the detection of markers of apoptosis illustrated that DSS administration increased the expression of BAX and the ratio of cleaved-caspase-3/caspase-3 but decreased BCL-2 expression in colon tissues. However, overexpression of HOXD10 obviously counteracted these effects (Figure 3c). Overall, these data indicated that HOXD10 alleviated DSS-induced UC in mice by suppressing apoptosis and facilitating proliferation.

### 3.4 HOXD10 inhibits DSS-induced inflammatory response and inflammatory pathway

**Figure 4:**
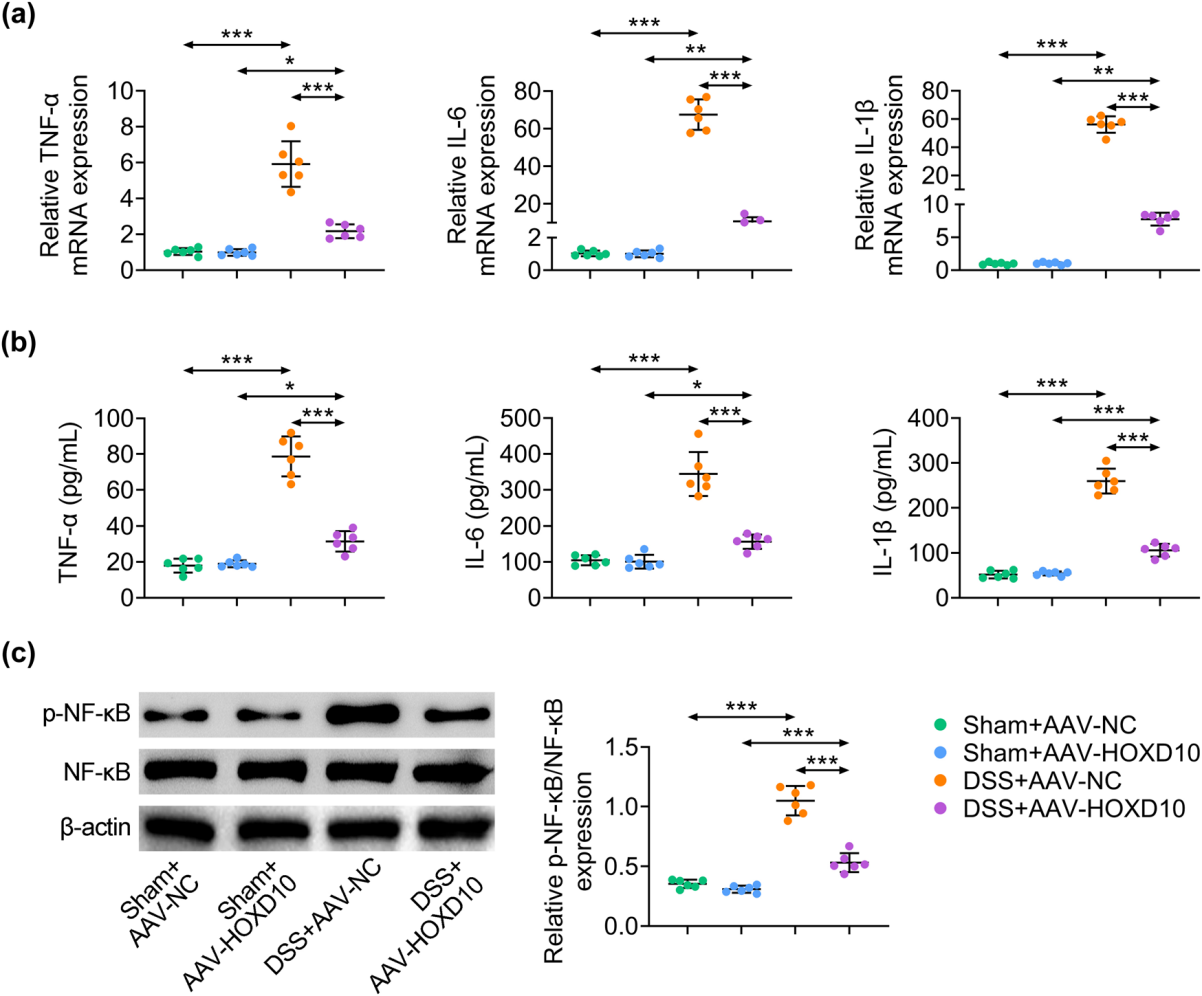
HOXD10 inhibits DSS-induced inflammatory response and inflammatory pathway. (a) The expression levels of TNF-α, IL-6, and IL-1β detected via qRT-PCR analysis (^***^ *p* < 0.001, ^**^ *p* < 0.01, ^*^ *p* < 0.05, C57BL/6 male mice, *n* = 6/group). (b) The expression levels of TNF-α, IL-6, and IL-1β detected via ELISA (^***^ *p* < 0.001, ^*^ *p* < 0.05, C57BL/6 male mice, *n* = 6/group). (c) The expression levels of NF-κB and p-NF-κB detected via western blotting analysis (^***^ *p* < 0.001, C57BL/6 male mice, *n* = 6/group). The one-way ANOVA with Bonferroni post-test was introduced for the statistical analysis in multiple groups.

Previous studies revealed that DSS-induced UC enhanced the production of proinflammatory factors and activated inflammation-related signaling pathway [29]. Thus, the expression levels of TNF-α, IL-6, and IL-1β in serum were determined by qRT-PCR and ELISA assays, and the results indicated that they were dramatically up-regulated in DSS-challenged mice. As expected, pre-treatment of HOXD10 effectively decreased the expression of inflammatory cytokines (Figure 4a and b). In addition, the p-NF-κB expression and the ratio of p-NF-κB/NF-κB increased by DSS administration in colon tissues were markedly reduced by HOXD10 overexpression (Figure 4c). These results suggested that HOXD10 significantly suppressed inflammatory response in DSS-induced UC.

### 3.5 HOXD10 reverses the intestinal permeability and improves intestinal barrier function

**Figure 5:**
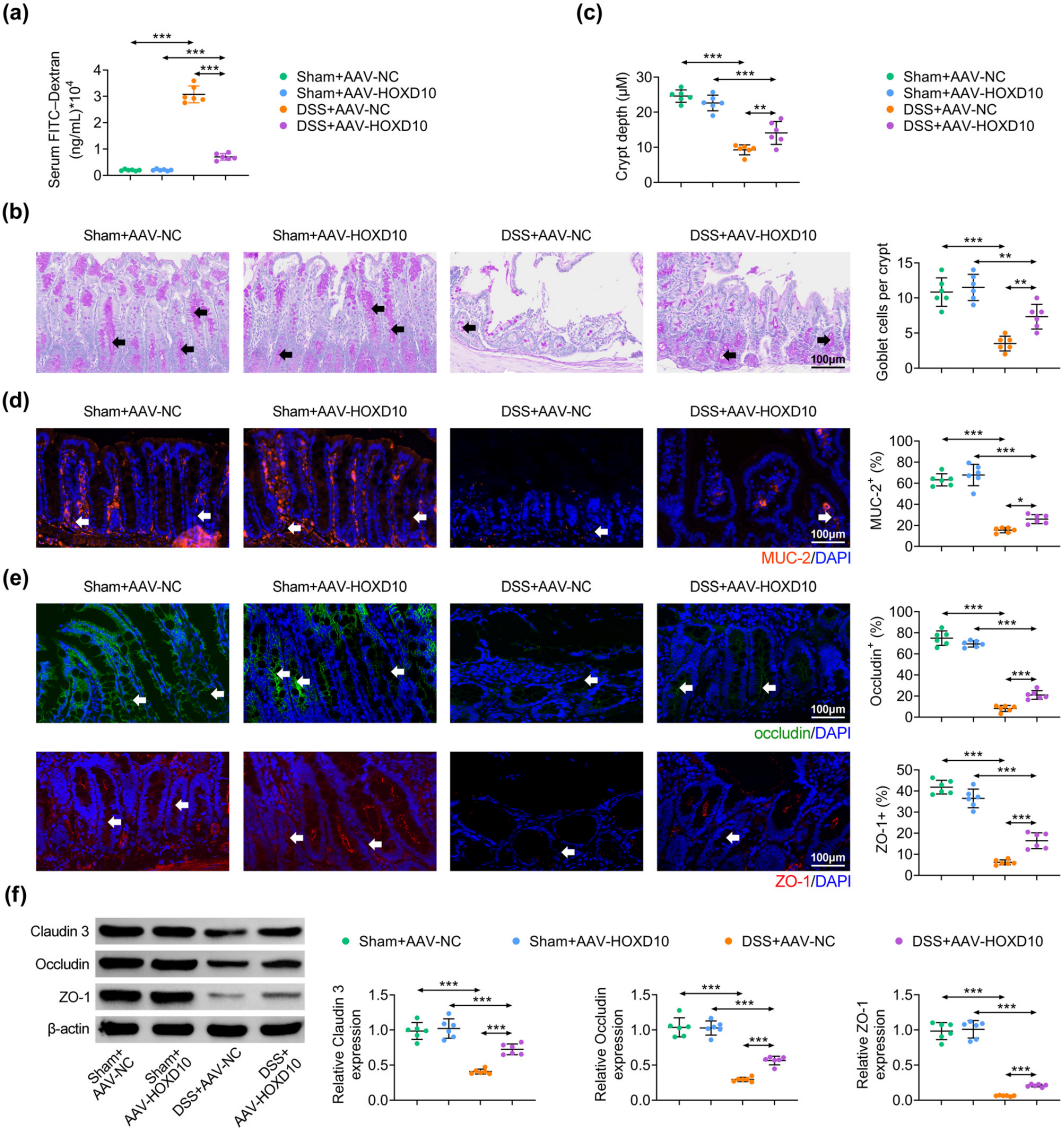
HOXD10 reverses the intestinal permeability and improves intestinal barrier function. (a) The content of FITC–dextran in serum (^***^ *p* < 0.001, C57BL/6 male mice, *n* = 6/group). (b) The goblet cells loss detected via PAS staining (^***^ *p* < 0.001, ^**^ *p* < 0.01, C57BL/6 male mice, *n* = 6/group). (c) The quantitative data of crypt depth (^***^ *p* < 0.001, ^**^ *p* < 0.01, C57BL/6 male mice, *n* = 6/group). (d and e) The expression levels of MUC-2, occluding, and ZO-1 detected via immunofluorescence analysis (^***^ *p* < 0.001, ^*^ *p* < 0.05, C57BL/6 male mice, *n* = 6/group). (f) The expression levels of Claudin3, occluding, and ZO-1 detected via western blotting analysis (^***^ *p* < 0.001, C57BL/6 male mice, *n* = 6/group). The one-way ANOVA with Bonferroni post-test was introduced for the statistical analysis in multiple groups.

Intestinal barrier dysfunction contributes to the up-regulation of FITC-dextran [8]. As shown in Figure 5a, DSS treatment dramatically elevated the content of FITC-dextran in serum compared with sham mice. Nevertheless, overexpression of HOXD10 effectively reduced the production of FITC-dextran (Figure 5a). Then PAS staining was performed to investigate the number of mucin-filled goblet cells and the morphology of intestinal crypts. The results showed that DSS administration obviously reduced the number of goblet cells and crypt depth in colon tissues, while pre-treatment with HOXD10 effectively attenuated these effects (Figure 5b and c). Since the vital components of the intestinal mucus were secreted via goblet cells, MUC-2 (in colon tissues) was significantly decreased in DSS-treated group and was recovered after AAV-HOXD10 pre-treatment (Figure 5d). Epithelial tight junction has been discovered to be crucial for maintaining the integrity of epithelium and inhibiting inflammatory response [8]. Hence, the effects of HOXD10 on proteins (Claudin 3, Occludin, and ZO-1) associated with tight junction in colon tissues were determined. Immunofluorescence assay demonstrated that the expression levels of Occludin and ZO-1 in colon tissues were obviously reduced in DSS-induced mouse model group but were effectively recovered by overexpression of HOXD10 (Figure 5e). Consistently, western blotting analysis confirmed that DSS administration decreased the tight junction proteins’ expression in colon tissues, which was notably restored by HOXD10 overexpression (Figure 5f). Taken together, these findings implied that HOXD10 ameliorated DSS-injured intestinal barrier function by restoring intestinal permeability and tight epithelial junctions.

### 3.6 HOXD10 inhibits the activity of Rho/ROCK/MMPs axis

**Figure 6:**
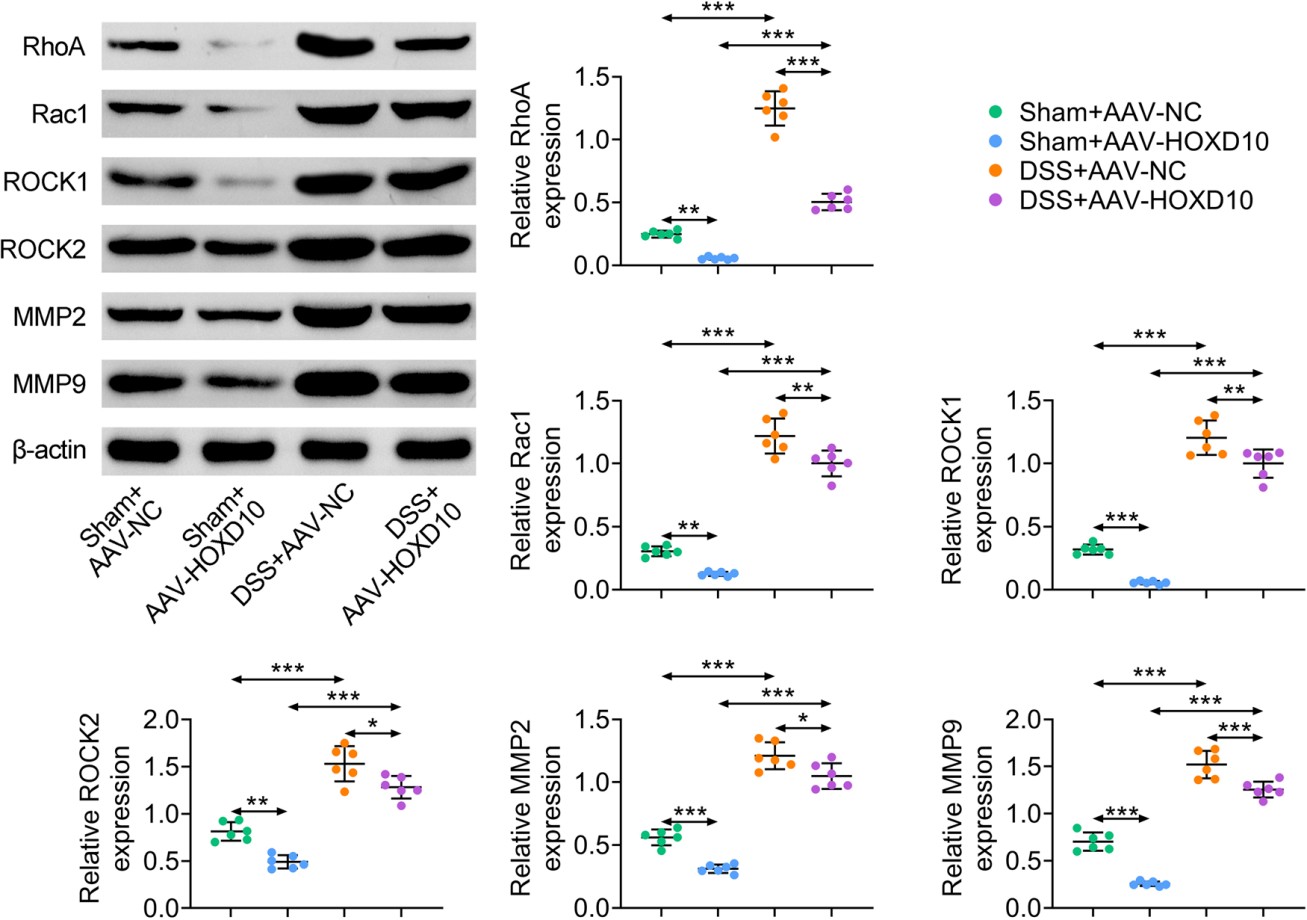
HOXD10 inhibits the activity of Rho/ROCK/MMPs axis. The expression levels of RhoA, Rac1, ROCK1, ROCK2, MMP2, and MMP9 detected via western blotting analysis (^***^ *p* < 0.001, ^**^ *p* < 0.01, ^*^ *p* < 0.05, C57BL/6 male mice, *n* = 6/group). The one-way ANOVA with Bonferroni post-test was introduced for the statistical analysis in multiple groups.

It has been reported that HOXD10 is able to regulate Rho/ROCK axis [10,11] and the inactivation Rho/ROCK pathway is involved in the DSS-induced epithelial barrier dysfunction, oxidative stress, and inflammatory reaction [13]. Additionally, emerging evidence verified that the members of MMP family exert essential roles in the UC progression and the Rho/ROCK inactivation down-regulates MMPs expression in hepatocellular carcinoma [14–16]. Therefore, we further explored whether HOXD10 modulates Rho/ROCK/MMPs axis in the development of DSS-induced UC by determining the expression level of proteins related to Rho/ROCK/MMPs axis (RhoA, Rac1, ROCK1, ROCK2, MMP2, and MMP9). As shown in Figure 6, DSS treatment notably increased the protein level of RhoA, Rac1, ROCK1, ROCK2, MMP2, and MMP9 in colon tissues, whereas HOXD10 overexpression effectively attenuated these effects. Collectively, these results revealed that HOXD10 suppressed the activity of Rho/ROCK/MMPs axis activated by DSS.

## 4 Discussion

This study revealed that HOXD10 was low-expressed in UC sample and was dramatically down-regulated by DSS treatment. In addition, overexpression of HOXD10 effectively alleviated DSS-induced UC symptoms, including the loss of weight, increase in DAI values, and shortened colon. Moreover, AAV-HOXD10 pre-treatment dramatically inhibited apoptosis and inflammatory response in colon tissues triggered by DSS. Moreover, HOXD10 overexpression recovered intestinal permeability, thereby improving DSS-challenged intestinal barrier function. Furthermore, HOXD10 suppressed the activation of Rho/ROCK/MMPs pathway in DSS-induced UC mouse model. Overall, these findings uncovered that HOXD10 might ameliorate DSS-induced colitis by mediating Rho/ROCK/MMPs axis.

As the main type of inflammatory bowel disease, UC has plagued many patients due to its high incidence and recurrence rate [30]. Previous studies have verified that a variety of factors including genes, environment, persistent neutrophil infiltration, and excessive production of pro-inflammatory factors initiate the progression of UC [31]. However, many therapies present irreversible side effects, including emesis, intoxication, systemic edema, and anemia [32]. Therefore, it is essential to explore new therapeutic targets and molecular mechanisms related to UC [33]. It has been reported that HOXD10 is a vital tumor suppressor that inhibits cells migration in various cancers [34–36]. In addition, increasing evidence has proved that HOXD10 is involved in inflammatory effects in diverse diseases such as rheumatoid arthritis [9] and Alzheimer’s disease [10]. Nevertheless, the detailed roles of HOXD10 in UC remain unclear. In this study, we analyzed the data of GSE48634 chip utilizing GEO2R analysis platform and discovered that HOXD10 expression was reduced in UC samples compared with healthy control samples. In previous studies, DSS-induced colitis in mouse is regarded as the well-known animal model for exploring the pathogenesis of UC. Therefore, DSS-induced mouse colitis model was introduced to mimic the clinical features of UC [32]. In line with the data from GEO database, the expression level of HOXD10 was notably down-regulated in DSS-treated mice as detected by qRT-PCR, western blotting, and immunofluorescence staining assays. These findings implied that HOXD10 may serve as a protective gene in UC patients.

Consistent with previous reporters, the clinical symptoms including weight loss, increased DAI value, and shortened colon were observed in model mice [37]. The obvious improvements of mice weight, DAI score, and colon length were observed by pre-treating mice with AAV-HOXD10. Moreover, overexpression of HOXD10 effectively restored the destruction of the colon epithelium and reduced the infiltration of inflammatory cells in the epithelium and lamina.

Recent studies verified that the imbalance of apoptosis and proliferation in colon is closely related to the pathogenesis of UC [38,39]. Consistently, we revealed that DSS administration notably inhibited proliferation and facilitated apoptosis in colon tissues, while HOXD10 overexpression effectively enhanced cell viability and suppressed apoptosis. Moreover, the excessive secretion of inflammation cytokines such as TNF-α, IL-6, and IL-1β and the phosphorylation of NF-κB have been found to affect the clinical symptoms of UC [40–46]. This study illustrated that the expressions of TNF-α, IL-6, and IL-1β at mRNA and protein level were remarkably reduced in AAV-HOXD10 group compared with DSS-treated group. In addition, HOXD10 overexpression suppressed NF-κB phosphorylation induced by DSS in colon tissues. These results demonstrated that HOXD10 was able to suppress inflammatory response in mice with UC.

The increased intestinal permeability is one of the leading characteristic of UC [47]. The mice in DSS-challenged group presented more serum FITC-dextran, suggesting the severe damages in intestinal mucosa. However, HOXD10 overexpression dramatically reduced the level of FITC-dextran, indicating that HOXD10 effectively ameliorated intestinal barrier.

The reduction of goblet cells is considered as an important feature of UC [8]. Our results revealed that DSS contributed to goblet cells loss and intestinal crypt distortion by employing PAS staining assay, while overexpression of HOXD10 effectively offset these effects. In addition, HOXD10 overexpression reversed the decrease in MUC-2 expression induced by DSS treatment. Moreover, previous study has proved that the intercellular tight junction is the vital guarantee for the integrity of intestinal mucus [8]. Tight junction proteins are considered to be the crucial component of the intestinal mucosal barrier, which reduce the space between intestinal epithelial cells and retain lumen material. These proteins such as Claudin 3, occluding, and ZO-1 are closely related to maintaining intestinal permeability [48]. It is reported that multiple drugs affect DSS-induced inflammation by regulating tight junction related proteins [49,50]. In line with previous reporters, in this study, DSS treatment decreased the expression level of Claudin 3, occluding, and ZO-1, whereas HOXD10 overexpression notably reversed these changes. These results proved that HOXD10 improved epithelial barrier dysfunction by eliminating DSS induced the decrease in goblet cells and MUC-2 and recovering the tight junction of adjacent epithelial cells.

Rho/ROCK axis has been reported to be closely associated with inflammatory response and epithelial barrier dysfunction [15] and HOXD10 is related to the inactivation of Rho/ROCK pathway [10,11]. Moreover, previous studies have demonstrated that the inactivation of Rho/ROCK suppresses the expression of MMPs and the members of MMPs are closely related to the pathogenesis of UC [17,18,20]. Therefore, we explored the effects of HOXD10 on Rho/ROCK/MMPs signals in experimental UC mouse model. Interestingly, DSS treatment significantly increased the expression levels of proteins related to Rho/ROCK/MMPs axis, which were obviously reduced by HOXD10 overexpression. These data confirmed that HOXD10 suppressed the activity of Rho/ROCK/MMPs axis in mice with UC.

However, this study is limited by the lack of direct evidence that the regulation of HOXD10 on UC processes is associated with the Rho/ROCK/MMPs axis. Further experiments are required to verify whether HOXD10 influences UC progression by mediating Rho/ROCK/MMPs axis and how HOXD10 inhibits the activation of Rho/ROCK/MMPs signaling.

## 5 Conclusion

In conclusion, this study for the first time elucidated that HOXD10 may effectively alleviate DDS-induced UC progression and suppress apoptosis and inflammatory response in mice by regulating Rho/ROCK/MMPs axis.
